# Regulatory T cells mediated immunomodulation during asthma: a therapeutic standpoint

**DOI:** 10.1186/s12967-020-02632-1

**Published:** 2020-12-02

**Authors:** Mohammad Afzal Khan

**Affiliations:** Organ Transplant Research Section, Comparative Medicine Department, King Faisal Specialist Hospital and Research Centre, Riyadh, Saudi Arabia

**Keywords:** Inflammatory cells, Airway inflammation, Regulatory T cells, Immunosuppression

## Abstract

Asthma is an inflammatory disease of the lung airway network, which is initiated and perpetuated by allergen-specific CD4^+^ T cells, IgE antibodies, and a massive release of Th2 cytokines. The most common clinical manifestations of asthma progression include airway inflammation, pathological airway tissue and microvascular remodeling, which leads to airway hyperresponsiveness (AHR), and reversible airway obstruction. In addition to inflammatory cells, a tiny population of Regulatory T cells (Tregs) control immune homeostasis, suppress allergic responses, and participate in the resolution of inflammation-associated tissue injuries. Preclinical and clinical studies have demonstrated a tremendous therapeutic potential of Tregs in allergic airway disease, which plays a crucial role in immunosuppression, and rejuvenation of inflamed airways. These findings supported to harness the immunotherapeutic potential of Tregs to suppress airway inflammation and airway microvascular reestablishment during the progression of the asthma disease. This review addresses the therapeutic impact of Tregs and how Treg mediated immunomodulation plays a vital role in subduing the development of airway inflammation, and associated airway remodeling during the onset of disease.

## Background

Asthma is an inflammatory disease of the lungs, which leads to wheezing and exacerbates breathing in the affected patients, and globally more than 339 million people are suffering, and more than 80% of asthma-associated fatalities occur in low-income countries and therefore new therapeutics are warranted to subdue and control asthma [1–8]. Pathologically, the progression of asthma is defined by airway epithelium hyperplasia, mucus cell metaplasia, increased airway smooth muscle mass, and increased deposition of extracellular matrix proteins, which disarray normal functioning of the lung airway system [9]. Airway inflammation is mediated by T cells, B cells, macrophages, eosinophils, and activated complement fragments, which play a critical role in tissue injury, and remodeling [6, 10–14]. Airway tissue associated remodeling phase refers to structural perturbations during asthmatic airway inflammation, and these perturbations are initiated and regulated through a series of cellular and molecular responses [15–20]. The conventional therapeutic formulations to control asthma have focused on the use of potent anti-inflammatory drugs, particularly steroids, which have broad-spectrum suppressive activity against effector cells and their mediators [21]. Management and control of asthma include inhaled corticosteroids, bronchodilators (β-agonists and anticholinergics), theophylline, leukotriene-receptor antagonists, leukotriene synthesis inhibitors, anti-IgE antibodies, anti-IL5 antibodies, and anti-IL4/IL13 antibodies [22–25]. Although, most popular glucocorticoid regimens are potent in most asthma patients but ineffective to support continuous respite of disease without repeated long-term administration, which can be associated with serious toxic side-effects, and fail to control the disease in a large number of asthma patients [26].

## Airway inflammation

Airway inflammation during asthma is characterized by the accumulation of Th2 type cells, IgE, and eosinophils, which leads to airway hyperresponsiveness and tissue remodeling. As a result, this inflammatory condition is related to a defective T cell immune response to various environmental allergens. Inflammation associated airway remodeling in lung diseases mainly represents structural changes associated with a reduction in lung functions, which includes sub-epithelial fibrosis, airway smooth muscle hypertrophy, hyperplasia, tissue eosinophilia, and epithelial injuries [11, 12, 15, 27–31]. These inflammatory responses are usually suppressed by Tregs, which maintain airway immunotolerance through IL-10, which is a vital immunosuppressive and antifibrotic cytokines secreted by the majority of regulatory cells [32–36]. During an immune response, Th1 cells produce IL-2 and IFN-γ; they are important in immune responses in allergic inflammation while Th2 cells are essential in allergic inflammation through IL-4, IL-5, IL-9, and IL-13 cytokines [1, 37–39]. Th1 cells secreted IFN-γ has inhibitory effects on Th2 cells, and during allergic inflammation, it suppresses isotype switching of IgE and it can also stimulate cell-mediated cytotoxic effects [1]. Furthermore, Th17 cells are a distinct lineage of Th cells expressing IL-17 and mediate neutrophilic type inflammation and exacerbate Th2 mediated-allergic inflammation [40, 41].

Clinical manifestations of asthma are characterized by airway inflammation, airway obstruction, airway hyperresponsiveness, and massive infiltration of eosinophils, neutrophils, T lymphocytes, and mast cells in the airway, which play a crucial role in the initiation and progression of chronic airway inflammation and airway remodeling [10, 12, 32, 39, 42–44]. The development and prevailing of asthma pathogenesis are highly modulated by both the inflammation of airways and mucosal injury inflicted by chronic inflammation. The activation of mast cells and eosinophils, and the subsequent release of leukotrienes (LTB4, LTE4, LTD4), cationic proteins (histamine), serine proteases, chemical inflammatory mediators, and cytokines make them crucial to lead the epithelium injuries [45, 46]. This results in the activation and release of fibrogenic cytokines (TGF-β1) to initiate the process of myofibroblast proliferation, which leads to the progression of subepithelial fibrosis, angiogenesis, smooth muscle hyperplasia, and mucus gland hypertrophy [47–49].

## Airway microvascular remodelling

Clinical studies have shown the role of Tregs in human asthma, but these studies have been hampered by the lack of a clear correlation between Tregs and airway microvascular remodeling, which is the main pathological symptoms of asthma [50, 51]. In healthy lungs, the airway microvasculature supplies key vital functions necessary for maintaining a normal physiological process [52]. In particular, it delivers oxygen and nutrients, and act as a primary site for most of the humoral immune response to foreign antigens, which confers the first line of immunity before the onset of disease. Microvascular remodeling during airway inflammation mainly triggers though the pro-angiogenic action of growth factors and inflammatory mediators, and as seen in both human asthma and allergic reaction that airway microvasculature affected during the progression of the disease, which further signifies the key involvement of microvasculature and airway remodeling during asthma [29, 53–55]. Previous investigations on airway microvascular remodeling in chronic airway inflammation demonstrated that microvascular components of airway remodeling are the vital contributors to the alteration of the airway wall in asthma and COPD (Chronic obstructive pulmonary disease) progression [43, 56]. Airway microvascular alterations as seen in patients with asthma are accompanied by a rise in airway blood flow and diminished β2-adrenergic vasodilator responsiveness, suggesting the presence of endothelial dysfunction, increased microvascular permeability and edema are common features during vascular remodeling in bronchial asthma [54, 57–59]. While most studies identify the immunosuppressive properties of FOXP3^+^ Tregs to control allergic airway inflammation, these studies do not explain any impact of Tregs in microvascular changes and associated remodeling, as reported in clinical conditions [60]. Increased microvascular permeability and edema are common features during vascular remodeling in bronchial asthma [57], however, most previous investigations on airway microvascular remodeling in chronic airway inflammation extracted clinical outcomes of patients with asthma, and these data demonstrated that microvascular components of airway remodeling are vital contributors to the alteration of the airway wall in asthma progression [15, 43, 54]. Interestingly, these airway microvascular perturbations are also seen during the development of COPDs [56]. Most of the ongoing therapies target the suppression of inflammatory response without modulating the actual pathogenic mechanism. Although glucocorticoids are the first drug choice to subdue airway inflammation, glucocorticoid treatment is also associated with the expression of IL-10, FOXP3 (Forkhead box P3) mRNA, and induction of Tregs in bronchoalveolar lavage (BAL) of asthmatic patients [61–63]. These observations speculated that the presence of Tregs in BAL is crucial to play an immunoregulatory role in mediating the suppressive effect of corticosteroids [64–67]. In the last decade, several therapeutic alternatives for asthma cure have been acquired; however, their selectivity limits their success because asthma pathology is a multifactorial event. Altogether, these airway microvascular changes in asthma and COPD are strongly associated with airway inflammation and contribute to an increase in airway wall thickness, which is associated with disease progression [54].

### Regulatory T cells

Various T cells have the potential to mediate targeted immunosuppression, but FOXP3^+^ Treg has emerged as a dominant cell type; they are involved in maintaining tolerance during asthma inflammation [68]. Tregs are potent immunosuppressive cells that are key in maintaining the homeostatic balance during dysregulated immune responses, which is a critical feature of asthma inflammation [69–74]. Tregs are generated in the thymus as a functionally mature T cell subset and in the periphery of naive T cells and are crucial in maintaining immunological unresponsiveness to self-antigens, and suppressing heightened immune responses destructive to the tissue during asthma inflammation [12, 69, 75–83]. Tregs play a vital role in modulating and regulating immune responses by establishing the phase of immunotolerance and negating toxic inflammatory reactions, which are essential to maintain routine tissue repair as seen in several preclinical and clinical studies [60, 62, 84]. Tregs (CD4^+^/CD8^+^) are characterized by intracellular expression of FOXP3, and mainly secrete various key regulatory cytokine, which includes IL-10, TGF-β to suppress heightened immune responses, and trigger inducible Treg expansion [85]. FOXP3^+^ natural Tregs and peripheral induced Tregs are key in maintaining immunotolerance against mucosal injury, pathogenic alloimmunity, diabetes, and facilitate tolerance induction in murine models of organ transplantations [77, 81, 86–88].

Treg regulates immune responses through the release of key regulatory cytokines, IL-10, and TGF-β and modulates inflammation [68, 89]. Treg-mediated immunosuppression mainly operates through the secretion of suppressive soluble factors (IL-10, TGF-β, IL-35, Fibrinogen-like protein 2, CD39, and CD73), cell contact-mediated suppression (through Galactin-1, CTLA-4, LAG-3), and competition for growth factors (e.g. IL-2) [90]. The clinical demand for Treg cell-based immunotherapy is rapidly rising, and different Treg subsets including natural Tregs, induced Tregs, CD8^+^ Treg cells, and regulatory cells has been reported in various preclinical and clinical studies of asthma, which highlighted a crucial link of airway inflammation, airway remodeling and associated immunosuppressive roles of regulatory T cells to counter the ongoing cellular and tissue dysfunctions [86, 91, 92].

Immunoregulatory therapies that balance from Th2 to Th1 paradigm have also been investigated but with limited success in clinical trials [93], and in numerous mouse models to investigate the immunological mechanism of asthma pathogenesis [94, 95], which further highlighted a modulatory role of regulatory cell mechanism to negate the inflammatory effects of most of the inflammatory cells [12]. Furthermore, several research investigations highlighted the cellular and molecular basis of Treg development and functions and implicate Treg dysregulation in major pulmonary diseases, including asthma [96]. The role of Tregs in asthma is scanty, and quite a few studies have reported their clinical benefits, which show that depletion of FOXP3^+^ Tregs augments, whereas the reconstitution of Tregs subdues lung allergic responses and in some studies of airway hyperresponsiveness (AHR) [97–99]. Alternatively, Treg depletion before sensitization is proven sufficient to augments the severity of inflammation, and AHR in the lung [100]. Adoptive transfer of Tregs has been proven sufficient to subdue inflammation before the start of tissue inflammation and microvascular repair [77, 79]. These studies emphasized that the reconstitution of antigen-specific FOXP3^+^ Tregs was found to subdue allergic inflammatory responses and hyper reactivity via the IL-10 dependent pathway [33, 81, 101], and further downregulated established inflammation and prevent airway remodeling when injected after disease onset [102]. The therapeutic application of Tregs has been tested in clinical and preclinical platforms to achieve desired immunosuppression and to suppress asthma inflammation [89, 102–107].

### Immunosuppression

Tregs have been reported in suppressing Th2 mediated immune responses to allergens and subdue allergic inflammatory conditions, and numerous preclinical studies have shown that the adoptive transfer of antigen-specific Tregs subdues the onset and progression of asthma in mice [84, 99, 101, 108]. Generally, Tregs prevent the generation of immune responses to self-antigens and other foreign antigens, including allergens, also limit immune responses to pathogens, protecting tissue from severe injuries [81]. Tregs modulate Th2-mediated lung inflammation, and their therapeutic potential is best described by evidence that therapies with Treg in allergic and asthma disease are associated with the induction or restoration of Treg function, e.g. glucocorticoids, allergen immunotherapy [63]. Tregs mediated immunosuppression has the potential to protect against allergic inflammation and asthma pathogenesis [92, 108, 109]. The primary immunosuppressive and regulatory function of Tregs is to control immune responsiveness and regulate hyper-airway response [92, 110]. Tregs are involved in maintaining immunological unresponsiveness to self-antigens, inhibit alloimmune inflammatory responses [79], counteract self-reactivity, neutralizing killer T cells during an inflammatory phase [111], and more specifically contribute to suppressing worsened immune reactions, which is destructive to the airway epithelium and normal physiological outcomes.

**Fig. 1:**
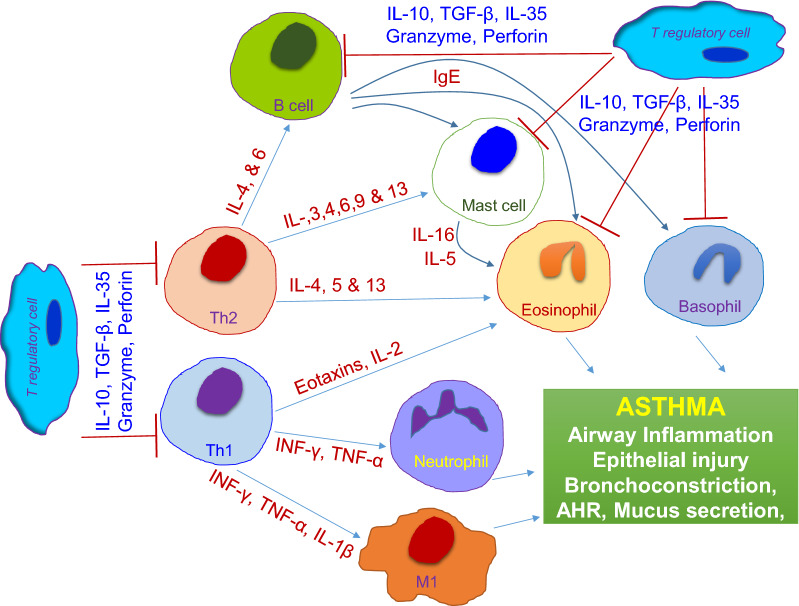
Overview of the immune system activation during asthma inflammation. Activation of Th1, Th2, B cells, Mast cells, Eosinophils, Neutrophils, Macrophages to promote inflammation, and associated tissue injuries, while Regulatory T cell mediated immunosuppression to check the ongoing inflammatory response

Treg operates through various immunosuppressive functions that regulate T lymphocyte, antigen-presenting cell, and innate cell functions through cell-contact, competition for essential growth factors, cytotoxicity [92, 96]. During allergic inflammation, Tregs mediate immunosuppression through the release of inhibitory cytokine IL-10, TGF-β, or by cell surface molecules [110, 112]. IL-10 mainly suppresses the effects of pro-inflammatory cytokines, restores epithelial layer integrity, tissue healing, and inhibits the survival and migration of eosinophils during allergic inflammation. IL-10 also down-regulates IL-4 induced isotype switching of activated B-cells [36, 108, 113]. Besides, Tregs have been associated with the maintenance of immune responses, and secreted immunosuppressive cytokines such as TGF-β, IL-10, and IL-35 are involved in immune responses following antigens/allergen exposure [114, 115] (Fig. 1).

In addition to the cytokine-mediated suppressive activity, Tregs are also mediate suppressive functions through the release of perforin and granzymes B and the release of cyclic adenosine monophosphate (cAMP) [90]. However, some clinical studies also validated these roles when treatment with glucocorticosteroids in asthmatics might increase this FOXP3 protein expression within Tregs in humans, and revealed the suppression of Tregs number as reported from lung tissue in a model of asthma [63] while asthmatic patients have been reported to show decreased FOXP3 protein expression within their CD4^+^ CD25^high^ T regulatory cells repertoire [116]. Data collected from patients with asthma further highlighted the crucial role of Treg, which reported lower Tregs ratio and FOXP3 mRNA expression, and lower levels in peripheral blood mononuclear cells may be associated with asthma pathogenesis in humans [117]. A significant number of murine models of allergic inflammation/asthma have been adopted, although none replicates all pathological parameters of human asthma conditions [94, 118]. However, studies in animal models of allergic airway inflammation have investigated a fair amount of preclinical and clinical research, which included the key roles of FOXP3^+^ Treg, IL-10, and TGF-β in asthma prevention [108]. In other clinical studies, adoptive transfer of purified antigen-specific FOXP3^+^Treg cells in pre-sensitized mice suppressed AHR, eosinophil recruitment, and Th2 cytokine release through the release of IL-10 and TGF-β, while the depletion of CD25^+^ Tregs before an allergen challenge shifted Th2 cytokine upregulation, IgE levels, eosinophilia, and AHR in allergy-resistant mice (C3H strain), concluded that Treg control disease resolution [99, 100, 119]. Altogether these previous investigations proved the therapeutic value of Treg to resolve established allergen-induced pulmonary inflammation (eosinophilia, Th2 infiltration, IL-5, IL-13, and TGF-β, but also prevent the progression of airway remodeling, and reduce mucus hypersecretion and peribronchial collagen deposition [99, 100]. Treg secreted IL-10 is a key anti-inflammatory and immunoregulatory cytokine that has distinct pleiotropic effects on both innate and adaptive immunity [120]. Primarily, it restrains inflammation and immune response and extensively participates in immunity activities by regulating cell proliferation, differentiation, and the function of T cells, B cells, macrophages, and endothelial cells [108]. IL-10 is produced by FOXP3^+^ Tregs and is also secreted by B cells, natural killer cells, antigen-presenting cells (APCs), mast cells, granulocytes. IL-10 can subdue the release of major pro-inflammatory cytokines such as IFN-γ, IL-2, IL-3, and TNF-α produced by Th1 cells, activated T helper cells, mast cells, NK cells, endothelium, eosinophils, and macrophages [33, 108]. Further, IL-10 can modulate Tregs to conserve the intracellular expression of FOXP3 and suppressive functions [121, 122]. IL-10 has wide immunosuppressive and anti-inflammatory properties suitable to attenuate asthma pathology [123, 124]. It is a powerful inhibitor of major proinflammatory cytokines and acts on antigen-presenting cells to subdue T lymphocyte activation (Th2), suppresses effector cells, mast cells, and eosinophils [33, 108, 114]. In addition, IL-10 augments IgG4 release, which plays a key protective in allergic responses but inhibits IgE [91]. Many clinical studies have reported higher IL-10 in allergic and asthmatics compared to healthy individuals [125]. IL-10 has been involved in effective immunosuppression of allergic immune reactions in the lung [101, 104, 126], which signifies dependence on IL-10 and further highlights the T regulatory cell-mediated modulation of pulmonary immune responses. These preclinical reports validated the key role of Tregs during airway remolding and disease progression, and key secreted anti-inflammatory cytokine-IL-10 play a vital role in airway allergic immunomodulation to maintain pulmonary physiological functions, and as reported, IL-10 suppresses Th1- and Th2-type immune responses, inhibits mast cells, eosinophils mediators, and pro-inflammatory cytokines [33, 108, 127]. Also, decreased IL-10 has been observed in allergic and asthmatic diseases compared with healthy control subjects [33]. Collectively, these regulatory networks are crucial to harness the reparative activity of Tregs, which could be an important therapeutic advantage in modulating allergic inflammation [90].

## Conclusions

Tregs mediated immunotherapy is a relatively new addition in modern drug development and therapeutics, and tend to replace conventional immunotherapy without negligible side effects in various inflammation-associated diseases including asthma. Modern drug discovery plan is quickly drifting toward a biological mode of therapeutic agents, which involve cells and their unique products to rescue the disease with minimum side effects, and global research is now in a new era with the introduction of clinical trials investigating the safety and potential therapeutic role of Treg therapy to rescue asthma exacerbations. The multi-regulatory action of Tregs recognized them as a potential candidate to rescue the occurrence of progressive inflammatory modulations, and superiority over the current immunosuppressive regimen, which makes this approach more of therapeutic value and will significantly minimize the cost of current immunosuppression for future medicine. These facts inspire the need for more specific therapies with the potential to support long-term recovery without side effects, and immunotherapeutic based on the understanding of Treg response to the pathophysiology of asthma could have overwhelming benefits for the cure of patients with asthma. In this review, we discussed airway inflammation, remodeling and Treg mediated protection to the progression of asthma pathogenesis. Therefore, the therapeutic use of Tregs to target effector responses may be the key approach to modulate the underlying cause of asthma disease, and to harness the immunoregulatory potency is of utmost requirement in asthma.

## Data Availability

Not applicable.

## Abbreviations

AHR: Airway hyperresponsiveness
APC: Antigen-presenting cells
BAL: Bronchoalveolar lavage
CTLA4: Cytotoxic T-lymphocyte-associated protein 4
cAMP: Cyclic adenosine monophosphate
COPD: Chronic obstructive pulmonary diseases
FOXP3: Forkhead box P3
LAG-3: Lymphocyte-activation gene 3
LTB4/ LTE4/ LTD4: Leukotriene B4/E4 and D4
Tregs: Regulatory T cells
